# Multimodal Treatment of Bone Metastasis—A Surgical Perspective

**DOI:** 10.3389/fendo.2018.00518

**Published:** 2018-09-07

**Authors:** Henry Soeharno, Lorenzo Povegliano, Peter F. Choong

**Affiliations:** ^1^Department of Orthopedics, St Vincent's Hospital Melbourne, Melbourne, VIC, Australia; ^2^Department of Orthopedics, Singapore General Hospital, Singapore, Singapore; ^3^Clinica Orthopedica, Universita di Udine, Azienda Sanitaria Universitaria Integrata di Udine, Udine, Italy; ^4^Department of Surgery, University of Melbourne, Melbourne, VIC, Australia

**Keywords:** bone metastasis, metastases, metastatic, prophylactic surgery, multimodal, metastatic bone disease

## Abstract

Over the past decades there has been an increase in the incidence of cancer worldwide. With the advancement in treatment, patient survival has improved in tandem with the increasing incidence. This, together with the availability of advanced modern diagnostic modalities, has resulted in more cases of metastatic bone disease being identified. Bone metastasis is an ongoing problem and has significant morbidity implications for patients affected. Multimodal treatment strategies are required in dealing with metastatic bone disease, which include both surgical and non-surgical treatment options. In the multidisciplinary team, orthopedic surgeons play an important role in improving the quality of life of cancer patients. Surgical intervention in this setting is aimed at pain relief, restoration of function and improvement in functional independence. In selected cases with resectable solitary metastasis, surgical treatment may be curative. With the advancement of surgical technique and improvement in implant design and manufacture, a vast array of surgical options are available in the modern orthopedic arena. In the majority of cases, limb salvage procedures have become the standard of care in the treatment of metastatic bone disease. Non-surgical adjuvant treatment also contributes significantly to the improvement of cancer patient care. A multidisciplinary approach in this setting is of paramount importance.

## Introduction

The Scandinavian Skeletal Metastasis registry reported an 18% increase in the incidence of cancer over the past decade (1). This is thought to be the result of an increase in the incidence of cancer as well as the improvement in diagnosis. Bone metastasis carries significant morbidity for afflicted patients and negatively impacts their quality of life. Following the lung and liver, bone is the third most likely affected site in metastatic cancer (2). Breast and prostate carcinomas have the greatest tendency to metastasize to bone (65–75%), followed by thyroid (60%), lung (30–40%), and renal (20–25%) carcinomas (3–5). The spine and the pelvis are the sites most frequently affected by metastasis (6). Long bones, such as the humerus and femur are also common sites for metastases (4).

Through the advances of modern cancer treatment options, we see a general improvement in the longevity of cancer patients, and hence an increase in the risk of bone metastasis (7). The management of patients with metastatic bone disease requires a multidisciplinary approach to ensure thorough diagnostic workup and treatment planning. A multi-modal treatment strategy, which includes medical therapy, radiotherapy and surgery, is encouraged in order to optimize treatment outcomes. In the setting of metastatic disease, surgical treatment is aimed at alleviating pain, restoring functional independence, and improving the overall quality of life of patients (8).

In the current modern orthopedic surgery arena, complex reconstructive surgery is made possible with the availability of advanced implant technology. Through better understanding of biomechanics and tribology, as well as better implant manufacturing processes, orthopedic surgeons now have a wide array of reliable implant options. Extensive bony defects can be resected and reconstructed with modern modular endoprosthesis (9). Advanced implant technologies, including modern locking plates and intramedullary nails have provided treating surgeons with a more robust reconstructive option (10). In the setting of metastatic bone disease construct fixation should be stable and strong enough to allow patients to immediately weight bear. In this regard, the modern implant repertoire allows individualization of treatment and a more predictable outcome.

## Principles of management and indication for surgery

### Diagnosis

Bone metastases can be asymptomatic and often present as an incidental finding during initial staging investigations. In some cases, they may be detected later during follow up in the setting of adjuvant treatment. It is important to note that about 75% of patients with bone metastases present with pain, which warrants further workup (11). Pain in bone metastases is unfortunately nonspecific; although certain characteristics such as rest pain, night pain or activity-related pain may raise the index of suspicion and indicate the need for further workup.

Metastatic bone disease typically involves multiple sites, which makes diagnosis relatively straightforward. A solitary bone lesion in the setting of a known primary carcinoma, on the other hand, can present a significant diagnostic dilemma. In these cases it is safe to assume the possibility of a malignant primary bone tumor, unless proven otherwise.

Adams et al. (12) reported on the consequences and prevention of inadvertent internal fixation of primary osseous sarcomas. In their study, 8 patients assumed to have metastatic disease underwent internal fixation and were later found to have primary bone sarcoma. As a consequence, 6 out of the 8 patients underwent an amputation. They concluded that inadequate history taking, incomplete staging imaging studies and improper biopsy resulted in these unfortunate incidences. Catastrophic inadvertent intramedullary nailing of a malignant primary bone tumor carries with it significant morbidity, since the majority of patients in such cases will require a high amputation for local disease control (12).

### Investigations

#### Plain radiography (X-ray)

**Figure 1:**
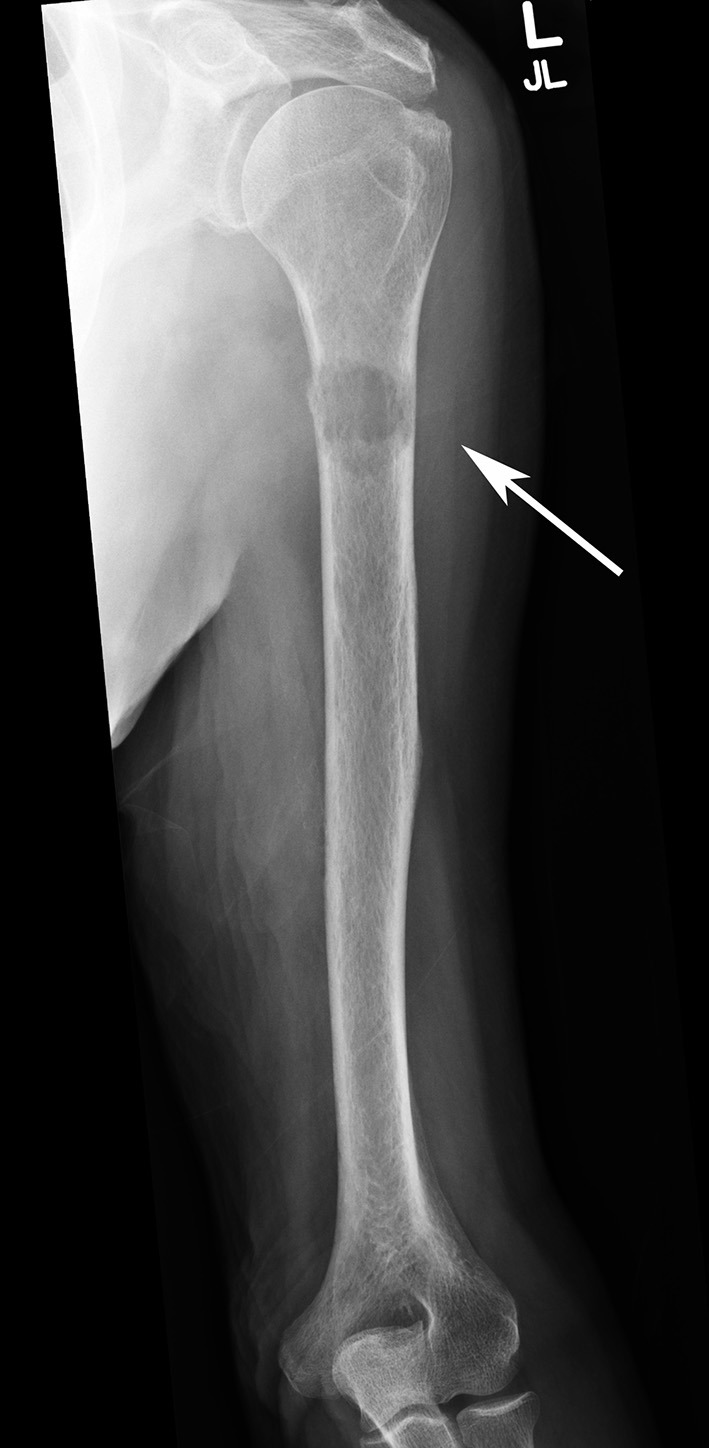
AP radiograph of a left humerus demonstrating a lytic metastatic lesion (arrow) in the proximal diaphysis. Note the extensive cortical involvement, predisposing it to a pathological fracture.

Orthogonal plain radiographs of the entire bone in question should be obtained, including the joint above and below. The radiographic appearances of metastatic lesions are usually described as osteolytic (Figure 1), osteoblastic, or mixed lytic-sclerotic (13). Prostate cancer classically gives rise to osteoblastic lesions, whereas renal carcinoma, lung carcinoma and multiple myeloma are osteolytic in appearance (13). Breast cancer often has a mixture of both lytic and sclerotic disease (13, 14). It is estimated that by the time a lesion becomes radiographically detectable, around 25–75% loss of bone mineral has occurred (15). For this reason, by the time a lesion is detectable on radiographs, the bone involved has weakened significantly (15, 16).

#### Computed tomography (CT scan)

**Figure 2:**
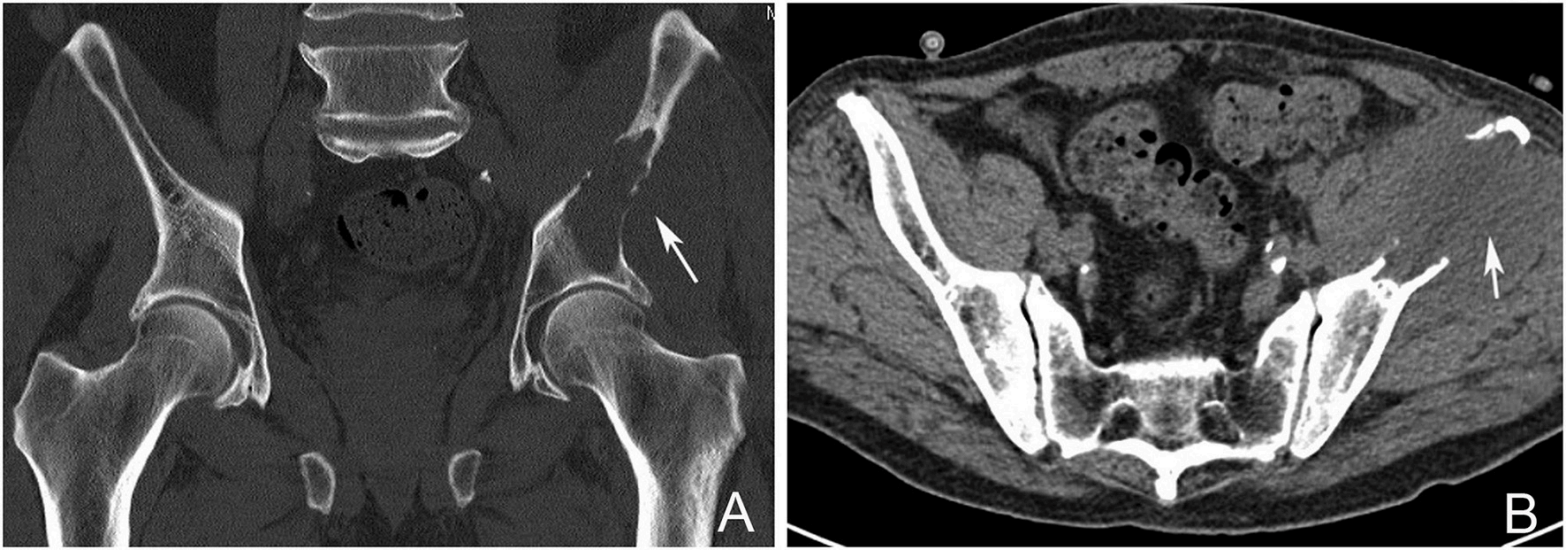
Pelvic CT Scan showing a left sided periacetabular renal cell carcinoma metastasis. **(A)** Involvement of the left supraacetabular region by a large lytic metastatic lesion (arrow). **(B)** Note the extensive extraosseous involvement (arrow).

CT scan is the most sensitive imaging modality available for evaluating the extent of cortical bone destruction (Figure 2) (17). It is also useful in image guidance during percutaneous biopsy of metastatic lesions. CT scan has a sensitivity of 74% and specificity of 56% in the detection of skeletal metastasis (18).

#### Magnetic resonance imaging (MRI)

MRI has a high sensitivity in detecting small metastatic lesions that are otherwise undetectable by other modalities such as CT scan and bone scan. Yang et al., in their meta-analysis comparing four imaging modalities (CT, MRI, FDG-PET, and bone scintigraphy) in the detection of bone metastases, found MRI to have a sensitivity of 91% and specificity of 95% (19).

**Figure 3:**
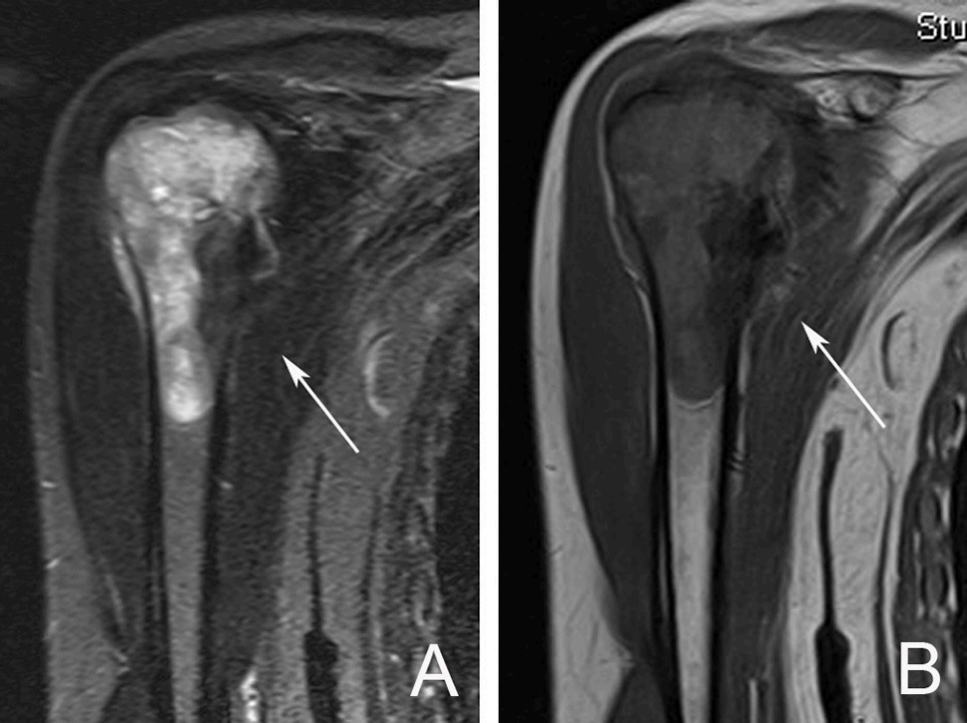
MRI scan demonstrating a right proximal humerus metastatic lesion. **(A)** T2 weighted MRI sequence showing the extent of intramedullary involvement (arrow). **(B)** T1 weighted MRI sequence showing complete involvement of the proximal humerus with cortical breach at the medial calcar region (arrow).

MRI is considered to be the most sensitive imaging modality for assessing the extent of intramedullary and extraosseous soft tissue involvement (Figure 3) (20). In the spine, the use of MRI allows the differentiation between osteoporotic and pathological fractures, since edema in osteoporotic compression fractures usually subsides by around 10–12 weeks (18).

#### Bone scan (99mTc bone scintigraphy)

**Figure 4:**
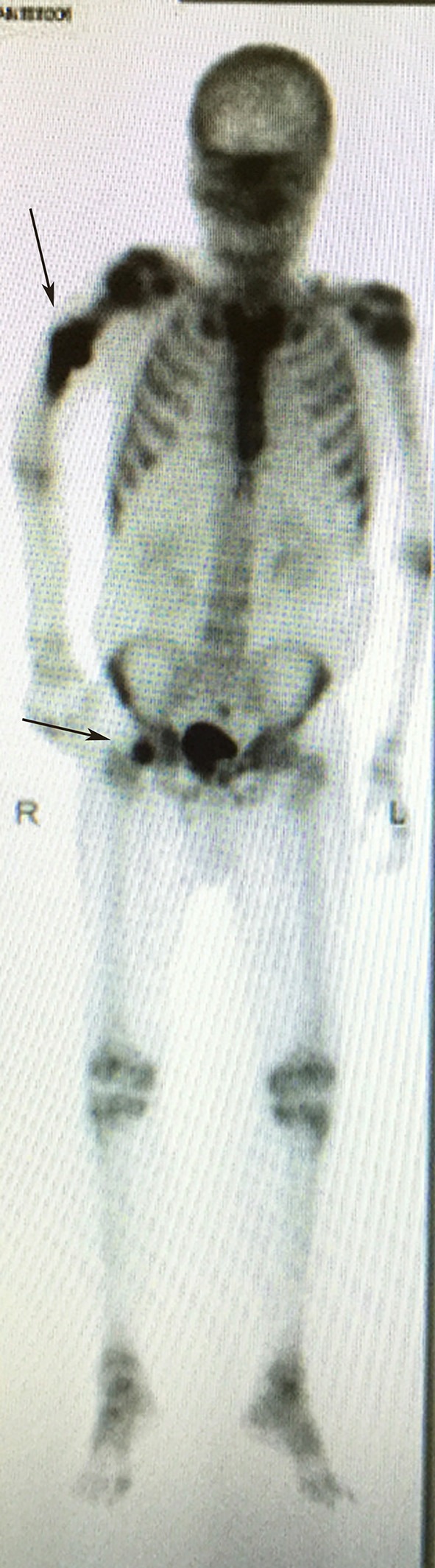
Bone scan demonstrating increase uptake at the right humerus diaphysis and right femoral head (arrows), highlighting the sites of bone metastasis.

Bone scan is a radionuclide-based imaging modality that measures osteoblastic activity and skeletal vascularity, hence its ability to detect osteoblastic metastases. It is also useful in determining whether a metastatic lesion is solitary or widespread, since the whole skeleton is captured during imaging (Figure 4). In rapidly growing lytic tumors, such as multiple myeloma, the bone scan may appear “cold” since minimal osteoblastic activity is present. In contrast false positive readings are common in areas with high bone turnover, such as seen in trauma and infection (21). The sensitivity and specificity of bone scan in detecting bone metastases has been reported as 78 and 48%, respectively (20).

#### Positron emission tomography (PET)

**Figure 5:**
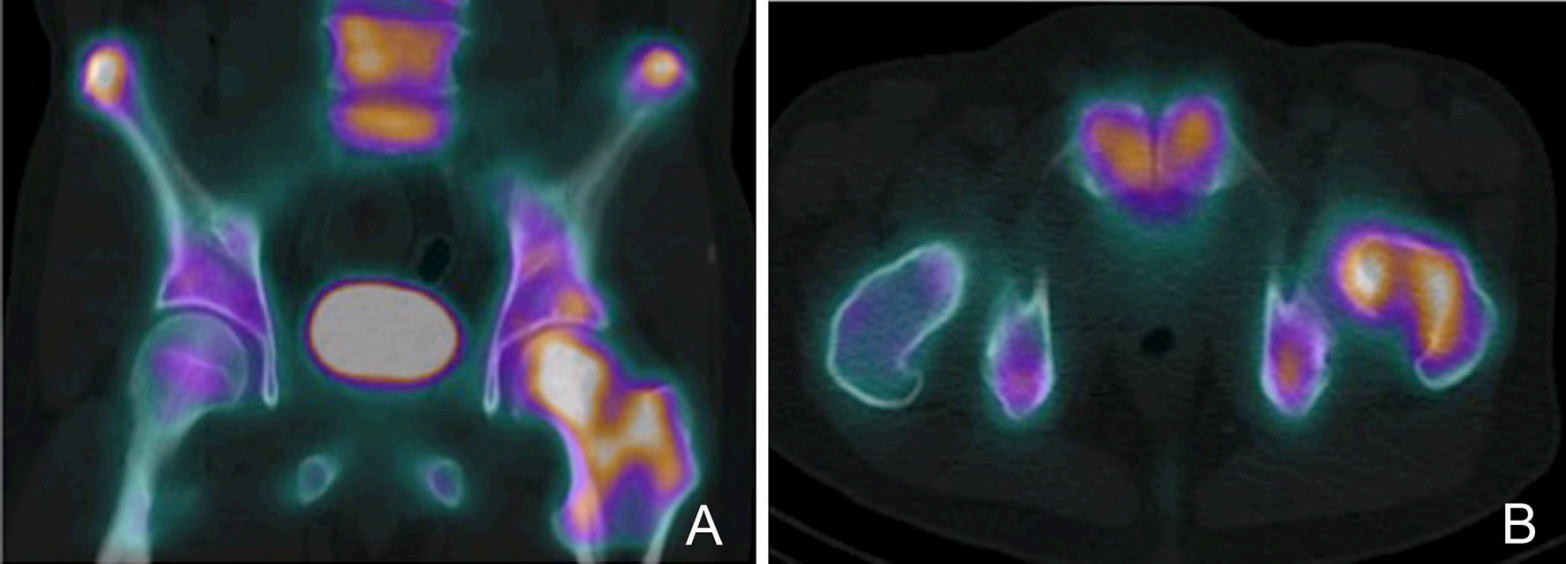
PET-CT scan demonstrating a left proximal femur metastatic lesion. **(A)** Coronal and **(B)** axial cuts of the PET-CT images demonstrating intense FDG uptake at the left femoral head, neck and intertrochanteric region.

Fluorodeoxyglucose-positron emission tomography (FDG-PET) is a nuclear imaging modality that detects the metabolic activity of tumors. It relies on the glucose uptake by tumor cells, hence its ability to detect early metastasis prior to any detectable bony destruction (20). Although highly sensitive (98%), FDG-PET on its own has low specificity (56%) since it is a functional rather than anatomic imaging modality (19). The combination of FDG-PET with anatomic imaging modality, such as CT scan, increases its specificity significantly (up to 97%) (Figure 5) (22).

### Tumor markers

Apart from routine blood testing, such as full blood count, renal and liver panels, tumor markers are used as part of the systemic staging process in cancer patients. Tumor markers are proteins that represent unique genetic signatures of a particular tumor histotype (Table 1), hence their role as diagnostic adjuncts. Tumor markers are also used in monitoring treatment response and in disease surveillance.

**Table 1:** Examples of commonly used tumor markers.

**Tumor marker**	**Disease**
CEA	Colorectal cancer
PSA	Prostate cancer
CA 15-3	Breast cancer
CA 125	Ovarian cancer
CA 19-9	Pancreatic cancer
Beta 2 microglobulin	Multiple myeloma

### Biopsy

Adequate tumor tissue is the key to diagnosis. Biopsies should only be undertaken after all other staging studies are completed. Biopsy may be taken intra-operatively during fracture fixation of a pathological fracture or as a staged procedure during the staging process. Core needle biopsy has been shown to be reliable and adequate for diagnosis in over 90% of cases (23–25). Image-guided core needle biopsy is usually utilized in order to accurately target the lesion and minimize the risk of a false negative reading (26). In areas that are difficult to access, such as the periacetabular area, percutaneous image-guided core needle biopsy has largely replaced the need for open biopsies. Since most impending or pathological fractures are non-emergency cases, surgical fixation should not be performed until a definitive diagnosis has been confirmed (12, 27).

### Prognosis and surgical decision-making

**Figure 6:**
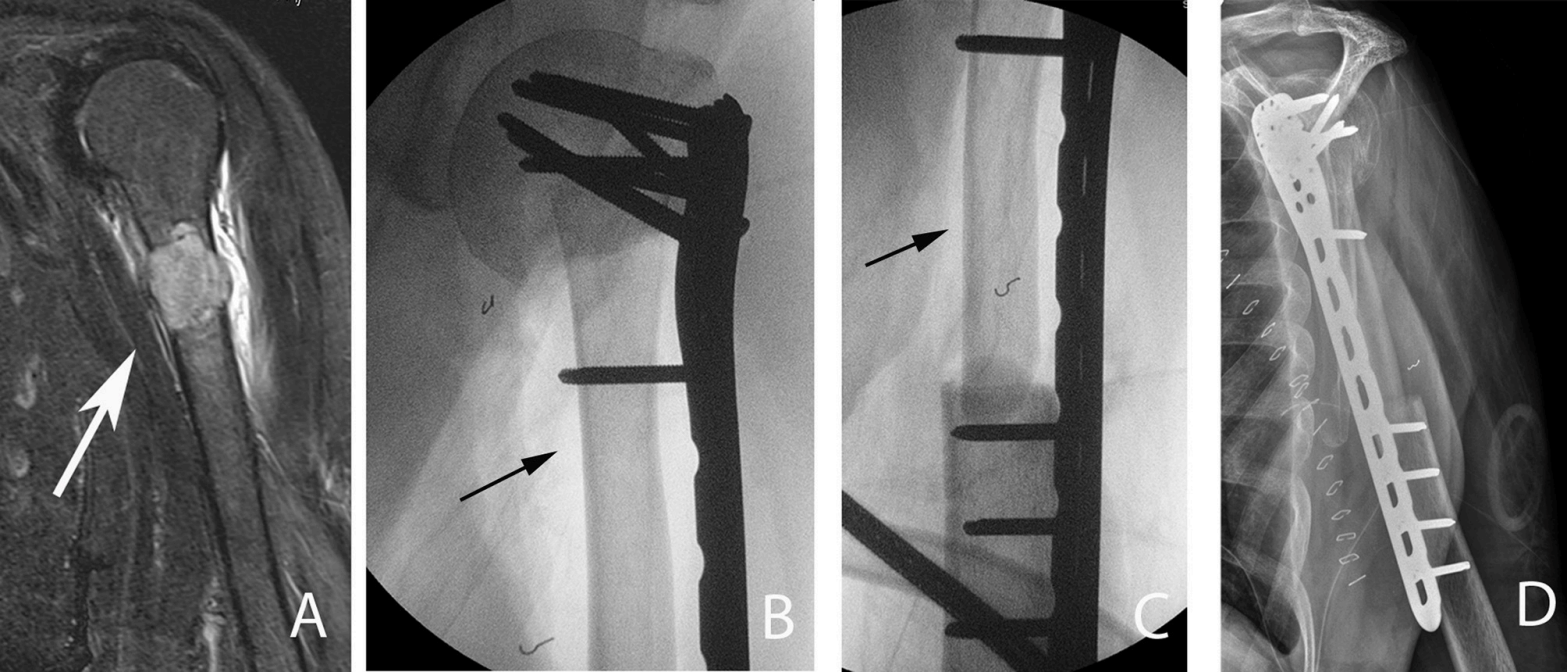
A 54-year-old patient with a left proximal humeral diaphyseal renal cell solitary metastasis treated with wide resection and reconstruction using a fibular allograft and locking plate internal fixation. **(A)** MRI of left humerus showing a metastatic lesion (arrow). **(B,C)** Intraoperative fluoroscopic images after intercalary resection of the proximal humerus diaphysis and reconstruction using a fibular strut graft (arrows). **(D)** Note the preservation of the native humeral head and the locking plate fixation.

The aim of surgical intervention in the setting of metastatic bone disease is to improve the quality of life of patients. Surgery allows pain control by achieving local control of the tumor, and at the same time, restoring the patient's functional independence. Following a thorough staging process to delineate the local and systemic extent of disease, a decision needs to be made as to whether treatment is aimed at palliative or curative intent. In the majority of metastatic conditions, surgical treatment is aimed at palliation, however, in selected cases such as resectable renal cell carcinoma with solitary metastasis, curative wide resection and reconstruction may be considered (Figure 6). Fottner et al. (28) in their retrospective review of 101 patients, who were treated surgically for skeletal metastasis of renal cell carcinoma, reported significantly better survival in patients with solitary metastatic lesions who underwent surgical wide resection. They also concluded that other factors contributing to higher survival include, age <65 years, absence of pathologic fractures and tumor-free resection margins.

Les et al. (29) in their retrospective review on 78 patients treated surgically for bone metastasis of renal cell carcinoma compared the rate of local progression between patients treated with local resection versus those who received intralesional procedures. Forty-one percent of patients in the intralesional procedure group required further procedures due to local progression. In contrast, only 1 out of the 37 patients who were treated with marginal or wide resection, required additional surgical intervention for local progression. They concluded that patients who receive intralesional procedures are at a much higher risk of local progression and therefore recommend surgical resection in order to minimize the risk of local progression.

The prognosis associated with a known primary cancer is a major deciding factor in determining the appropriate type of surgical treatment in metastatic bone disease. Longer survival is associated with an increased risk of disease progression or recurrence, hence more aggressive surgical treatment is often warranted. Kirkinis et al. (30) in their review on survival, prognostic factors, and outcomes after surgical treatment of appendicular skeleton bone metastases found several factors to be important predictors of prognosis. These include the primary tumor histoptype and the presence of visceral metastasis, pathological fractures, and multiple metastases. Patients with metastatic disease from renal cell and breast carcinoma were found to have the longest survival, whereas lung carcinoma and myeloma patients were shown to have the worse prognosis.

Given the numerous factors that contribute to the overall survival of patients, making a prognostic prediction is a major challenge. Over the years, several predictive tools have been designed to aid in the treatment decision-making process. Forsberg et al. (31) reviewed the Bayesian Belief Network (BBN) as a model for predicting patient survival. The model is designed to calculate the predicted survival at 3 and 12 months and subsequently guide surgical treatment options. They suggested that an estimated survival of <3 months does not support surgical treatment of impending pathological fractures. Patients with an estimated survival between 3 and 12 months were recommended for less invasive surgical management not associated with prolonged rehabilitation. When the predicted survival was more than 12 months, a more robust surgical option, such as tumor resection with endoprosthetic reconstruction was recommended.

Predictive models such as the BBN are invaluable in deciding the most appropriate surgical options, however the ultimate surgical treatment modality should be individualized for each patient. The general rule still applies, that any surgical fixation in metastatic bone disease should be sufficiently robust to allow early weight bearing while minimizing any potential complications. The type of fixation needs to have adequate durability to last patients for their remaining lifespan.

### Pre-operative planning

Careful pre-operative planning and the use of appropriate implants are fundamental in oncology surgery. Patients with malignancy should be managed by a multidisciplinary team, as these patients tend to be physiologically compromised and have elevated surgical risk. Meticulous coordination between multidisciplinary team members (medical oncologist, radiation oncologist, orthopedic surgeons, physiotherapist, nursing staff) is paramount in ensuring high quality care.

The role of surgery for bone metastasis can be divided into (i) prophylactic fixation to prevent impending pathological fractures, (ii) stabilization of a pathological fractures, (iii) segmental resection of tumors, and (iv) arthroplasty for replacing joints that have been destroyed by tumor. To this end, orthopedic surgeons have a vast array of surgical devices and implants in their surgical armamentarium at their disposal. These include plates and screws, intramedullary fixation devices, and tumor endoprostheses. The use of percutaneous intralesional injection of polymethylmethacrylate acid (PMMA) in osteoplasty, offers a minimally invasive management option for some contained tumors, e.g., vertebral metastases (4).

### Assessing risk of fracture

The definition of an impending pathological fracture remains ambiguous and it is the role of treating orthopedic surgeons to recognize them in a timely manner so that appropriate intervention can be administered. When a metastatic lesion has destroyed 30–50% of bone, usually it is deemed that a fracture is impending (32). Treatment strategies are strongly based on the risk of fracture and expected survival of the patient.

Several radiographic-based guidelines have been proposed in the past to aid in the decision-making regarding the need for prophylactic fixation. Fidler (33) proposed prophylactic fixation of long bones with more than 50% destruction by metastasis. Harrington (34) amended Fidler's guide, adding the criteria of: length of lesion of more than 2.5 cm, fractures around the femoral lesser trochanter region and persistent pain post radiation therapy. These guidelines, although useful, were somewhat oversimplified for actual clinical practice application.

In 1989, Mirels (35) developed a scoring system to predict the risk of impending fractures. This system offers a general guideline regarding when to intervene and remains one of the most widely system used. The Mirels scoring system (Table 2) is based on a point system that incorporates four criteria (nature of lesion, location, size of cortical involvement and pain), with each criteria carrying a score from 1 to 3 with increasing severity. Non-surgical treatment is recommended for scores of ≤ 7 and radiation therapy is usually considered as a means of local control. Scores >9 carry a strong recommendation for prophylactic fixation. Scores between 7 and 9 are open to debate as to whether surgery is indicated, and this is where institutional experience prevails. Despite being more comprehensive, the Mirels scoring system has some limitations. The amount of cortical destruction is determined based on two-dimensional orthogonal radiographs, which limits accuracy in the estimation of cortical involvement. The Mirels scoring system has low sensitivity and specificity, moreover, there is uncertainty regarding treatment for patients with a score of 8 (35).

**Table 2:** Mirels score.

**Score**	**Site**	**Pain**	**Lesion**	**Size**
1	Upper limb	Mild	Blastic	<1/3
2	Lower limb	Moderate	Mixed	1/3–2/3
3	Peritrochanteric	Functional	Lytic	>2/3

*Mirels score ≥ 9 High risk, 8 Intermediate, ≤ 7 Low risk for fracture*.

Nazarian et al. (36) developed and validated a CT-based rigidity analysis (CTRA) utilizing the quantification of changes in bone geometry and density. The system allowed for calculation of bone resistance to uniaxial loads, bending moment and torsional moment. In their multicenter prospective study, orthopedic tumor surgeons selected treatment plans for 124 patients with metastatic bone disease based on the Mirels scoring system. In the study, 36 patients had their treatment plan changed by their treating surgeon after CTRA results were provided. Their study concluded that CTRA had a sensitivity of 100% and specificity of 90% in predicting pathological fractures in comparison to the Mirels score (71% sensitive and 50% specific) (36).

The biology of pathological bone differs from that of normal bone. In pathological bone, the inherent ability to heal is impaired, hence most of these fractures require surgical fixation for stabilization (37). Standard fracture fixation techniques are often inadequate in dealing with pathological bone, hence rigid fixation techniques and strategies that account for the abnormal healing response and progressive nature of metastatic disease (locally and systemically) are required (38, 39).

In suitable cases, curettage of large lesions followed by cementing and supplementary plate fixation can provide a sufficiently robust construct to allow for early weight bearing. The ability to perform curettage on lesions prior to filling with bone cement allows for reduction in disease burden, which has been shown to reduce pain significantly (39). Leggon et al. (40) examined the torsional strength of canine femur bone that had simulated tumor defects treated with either bone cement and/or compression plating. Their result showed that the combination of bone cement and plating resulted in a construct that was 2.6 times stronger in torsional strength when compared to those with plate fixation alone (40).

### Bone metastasis by region and technical consideration

#### Long bones

Only around 10% of all skeletal metastasis affects the long bones as opposed to the axial skeleton, which accounts for up to 70% (41, 42). In long bone metastasis, the two most common sites are the proximal femur and proximal humerus (2). With the exception of lung carcinoma, metastatic carcinoma rarely affects areas distal to the elbows and knees (42). Due to its tendency to metastasize via the systemic arterial blood supply, lung cancer metastasis tends to be more widespread and may affect distant sites such as the hands and feet (43). Although the majority of bone metastases occur in the axial skeleton, most pathological fractures occur in the long bones (42).

Pathological fractures of the lower limb have a significant impact on a patient's mobility, whereas upper limb pathological fractures will greatly affect a patient's functional independence. Surgical management of lower limb long bone impending and pathological fractures is recommended as non-surgical management has been shown to have inferior results in controlling pain and restoring limb function (44).

**Figure 7:**
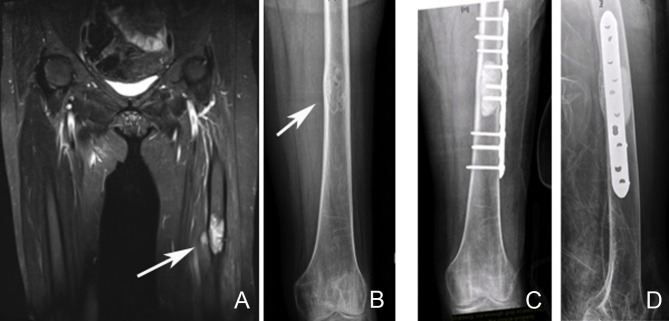
Left femoral diaphyseal metastatic lesion from breast carcinoma treated with curettage followed by cement-plate surgical fixation. **(A)** MRI showing a left femur diaphyseal intramedullary metastatic lesion. **(B)** The same lesion seen on plain X-ray. Note the mixed lytic sclerotic appearance of the lesion. **(C,D)** AP and lateral post-operative X-rays after curettage and cement-plate surgical fixation.

Various surgical options are available, such as internal fixation with extra or intramedullary devices to endoprosthetic arthroplasty options. Bone cement (PMMA) is frequently used to fill large bone defects in order to augment fixation constructs (45). It has the advantage of immediate stability due to its high compressive strength (Figure 7). The use of bone graft for void filling in metastatic disease is not usually recommended, since graft incorporation is less likely in post-irradiated bone (46, 47). Moreover, the effect of adjuvant chemotherapy delays graft healing and the shortened survival of patients with metastatic disease would make prolonged immobility of the limb, while waiting for the graft to heal, untenable (42).

The choice of fixation technique is largely guided by the location of the lesion, amount of bony involvement and disease response to systemic treatment (39). It is important to choose a fixation construct with the assumption that pathological bone will not heal and that a second revision surgery may not be tolerated by patients with metastatic bone disease. The construct of choice should be robust enough to allow immediate weight bearing for the likely survival time of the patient (48).

### Femur

The proximal femur is one of the most common areas for pathological fractures to occur. One third of such fractures occur at the femoral neck. Internal fixation of pathological fractures at the femoral neck generally results in an unfavorable outcome with high fixation failure rates due to poor healing potential of pathological bone (49).

Arthroplasty/endoprosthetic replacement procedures have a more reliable outcome in dealing with proximal femur pathological fractures, as it does not rely on bone healing which is necessary following treatment with internal fixation Steensma et al. compared failure rates between endoprosthetic reconstruction, intramedullary nailing and open reduction-internal fixation, in their retrospective study of 298 patients with proximal femur pathological fractures. They found that the endoprosthetic replacement group had a significantly lower failure rate (3.1%) when compared to the intramedullary nailing (6.1%) and open reduction-internal fixation (42.1%) groups (50).

In pathologic bone, the innate healing ability is impaired, which renders implant bony on-growth or in-growth unreliable. This healing impairment is even more significant in post-irradiated bone, hence cemented stem implants are recommended in this scenario (50). Cemented stems offer immediate stability while minimizing the risk of subsequent loosening. An important consideration is the use long-stem prosthesis in order to protect the remaining femoral shaft that may be affected by future metastatic deposits due to disease progression (51).

**Figure 8:**
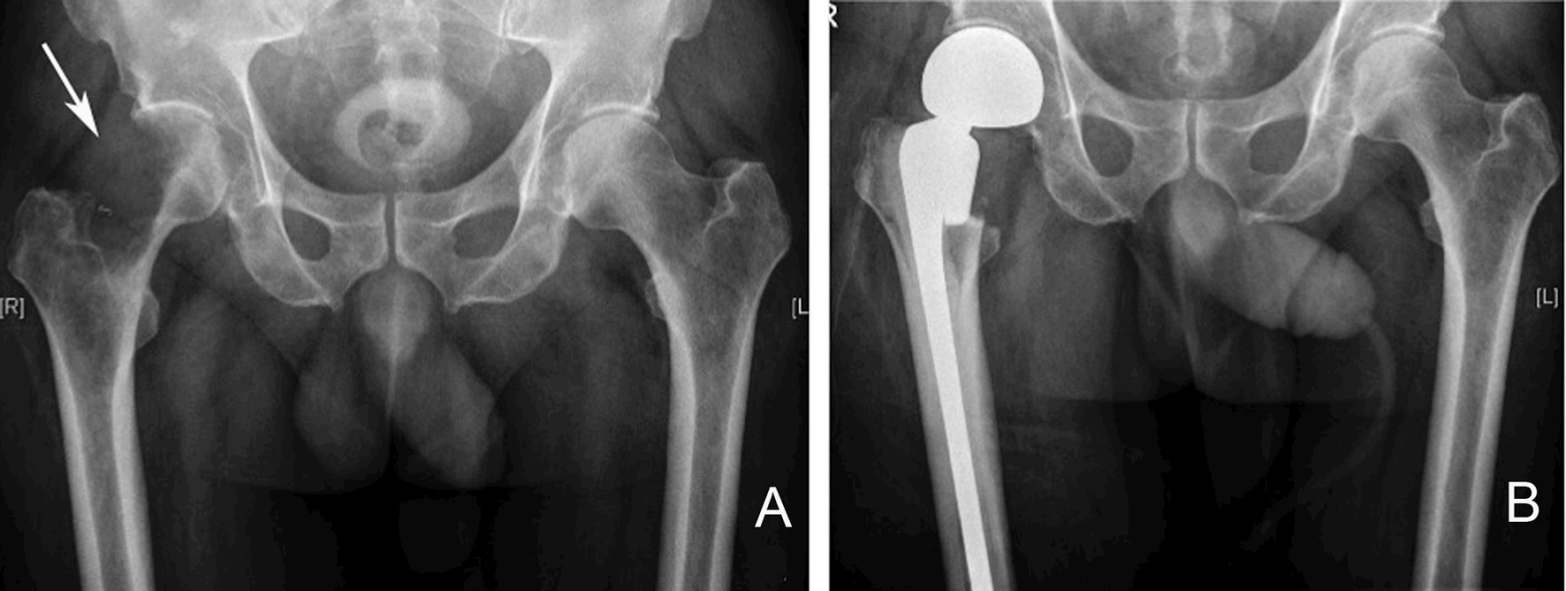
Right proximal femur metastatic melanoma treated with a calcar replacing hemiarthroplasty. **(A)** Large destructive lytic metastatic lesion involving the head and neck of the right femur. **(B)** X-ray post reconstruction with a calcar replacing hemiarthroplasty implant.

**Figure 9:**
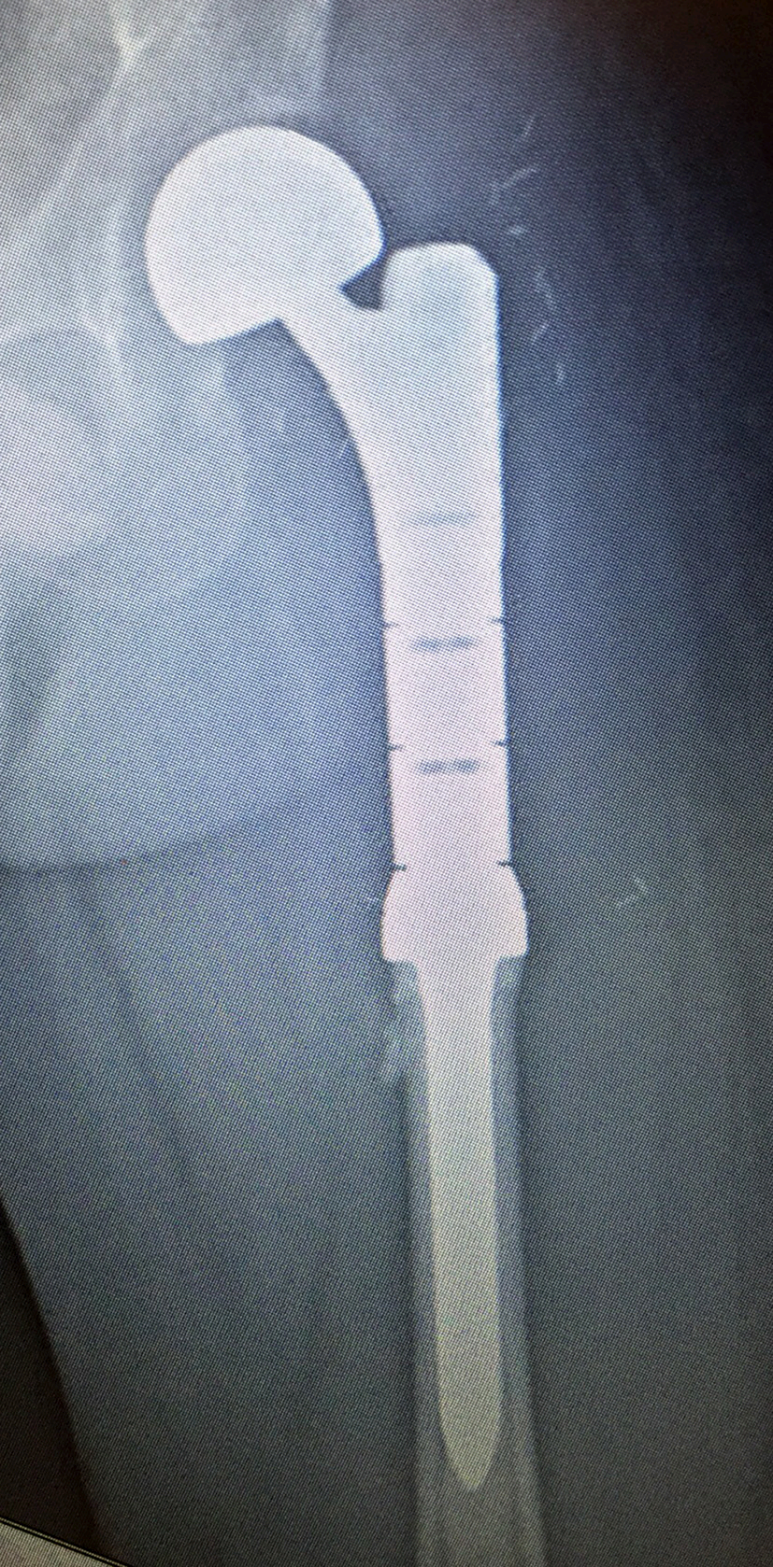
Reconstruction using a left proximal femur replacement endoprosthesis following resection. The modularity of these implants allow for accurate restoration of limb length.

The options of hemiarthroplasty and total hip replacement are both available, the choice of which depends on the presence of acetabular involvement. In cases where the acetabulum is spared, a hemiarthroplasty is adequate (50, 51). Involvement of the calcar femorale will necessitate the use of a femoral stem with a calcar replacing option (Figure 8). When there is extensive bony involvement, a proximal femur endoprosthesis is usually required (Figure 9). As a general rule, the femoral stem of the arthroplasty implant of choice should bypass the most distal aspect of the metastatic lesion by at least two cortical widths. This is to minimize the risk of subsequent periprosthetic fractures (52).

**Figure 10:**
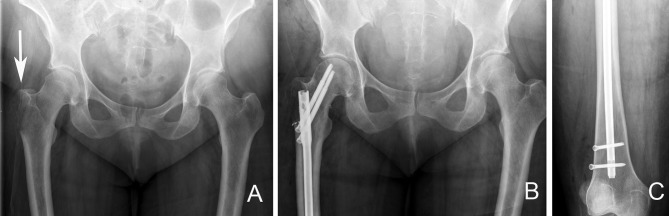
Right proximal femur bone metastasis treated with a locked intramedullary nail. **(A)**. Destructive lytic lesion involving the proximal femur greater trochanter area (arrow). **(B)** Postoperative X-ray after fixation with a cephalomedullary nail. Note the proximal fixation spanning the femoral head and neck. **(C)** Distal locking bolt fixation to ensure axial and rotational stability.

Peritrochanteric fractures or lesions may be addressed using plates and screws construct, such as a sliding hip screw, or a cephalomedullary device (Figure 10); the later has the advantage of being a load sharing device with superior biomechanical properties (53). Tanaka et al. in their retrospective study of 80 intramedullary nailing procedures for femoral metastases, reported implant survival rate of 94% at both 2 and 3 years. Three intramedullary nail implant failures occurred in those with subtrochanteric metastases (3 of 46 patients), which were subsequently revised with endoprosthetic reconstruction. They concluded that intramedullary nailing for femoral metastases is an adequate fixation method and allows for a less invasive method of fixation at a lower cost. They also emphasized that in the event of implant failure, endoprosthetic replacement is a viable salvage option (54).

Adjuncts such as PMMA (bone cement) may be used to augment the construct following tumor debulking via curettage. Since internal fixation in this region relies on bony purchase at the femoral head and neck region, it is important to rule out metastatic involvement in these areas preoperatively. In cases where there is involvement of the femoral head or neck, the use of proximal femur replacement endoprosthesis offers a more reliable solution (55).

Subtrochanteric and diaphyseal femoral involvement are most commonly addressed using locked intramedullary nails. Prophylactic fixation of impending pathological fractures is preferred, as fixation of an actual pathological fracture has been shown to result in inferior functional outcome and longer hospital stay. Arvinius et al. (56) in their retrospective study of 65 patients with metastasis to the femur, compared those who received surgical treatment prophylactically for impending fractures (21 patients) versus those who required treatment for pathological fractures (44 patients). All patients underwent fixation using a cephalomedullary device. In their study, 100% of patients who underwent prophylactic fixation for impending fracture were able to ambulate postoperatively, as compared to only 75.9% in the pathological fracture group. They concluded that patients who underwent prophylactic nailing required less postoperative blood transfusion, were able to ambulate earlier (day 4 vs. 9.7) and required shorter hospital stay (8 vs. 16 days) (56). Intramedullary nailing allows for a minimally invasive surgical approach, which minimizes intraoperative blood loss and surgical time significantly. This is particularly favorable in cases where patients are physiologically unfit to undergo lengthy surgical procedures.

The femoral subtrochanteric region undergoes tremendous amounts of stress during weight bearing, with loads up to 4–6 times body weight. Locked intramedullary nail spanning the whole femur with proximal fixation to the femoral head and neck is recommended (57). Careful perioperative workup and intraoperative monitoring is required to minimize the risk of pulmonary embolic phenomena, which may be life-threatening. Large subtrochanteric metastatic lesions may render intramedullary fixation inadequate since the implant are subjected to tremendous load-bearing stresses in such cases. This predisposes the implant to early failure and in these circumstances, proximal femoral replacement with a tumor endoprosthesis offers a more reliable solution (52, 57). Postoperative adjuvant radiotherapy should be given to the entire bone following fixation with a locked intramedullary nail, as soon as surgical wound healing has occurred (57).

Distal femur involvement by metastatic disease may pose a challenge in deciding the most appropriate implant choice due to its periarticular location. In cases where there is joint sparring with adequate bone stock, the use of curettage and PMMA augmented plate fixation or retrograde intramedullary nailing may provide adequate fixation (2). Ahmadi et al. (58) performed biomechanical testing on 15 synthetic femurs, comparing the mechanical stiffness and strength of retrograde nail, lateral locking plate and lateral non-locking plate. In their testing, a tumor-like defect was created at the lateral metaphyseal region, which was then filled with bone cement prior to fixation. Their results show that all three fixation types were similar in terms of axial stiffness, however retrograde nail was found to be superior to non-locking plates in terms of torsional and sagittal bending stiffness. They concluded that having the advantage of less soft tissue dissection, retrograde intramedullary nailing may be a sound option in dealing with distal femoral metastatic disease (58). It is important to note that their study was conducted using synthetic femur models which lacks the anisotropic property of biological bone. The other limitation of their study is that no comparison was made with retrograde nailing without bone cement augmentation. The addition of curettage and bone cement filling would somewhat negate the less invasive advantage that retrograde nail has over other open fixation methods.

**Figure 11:**
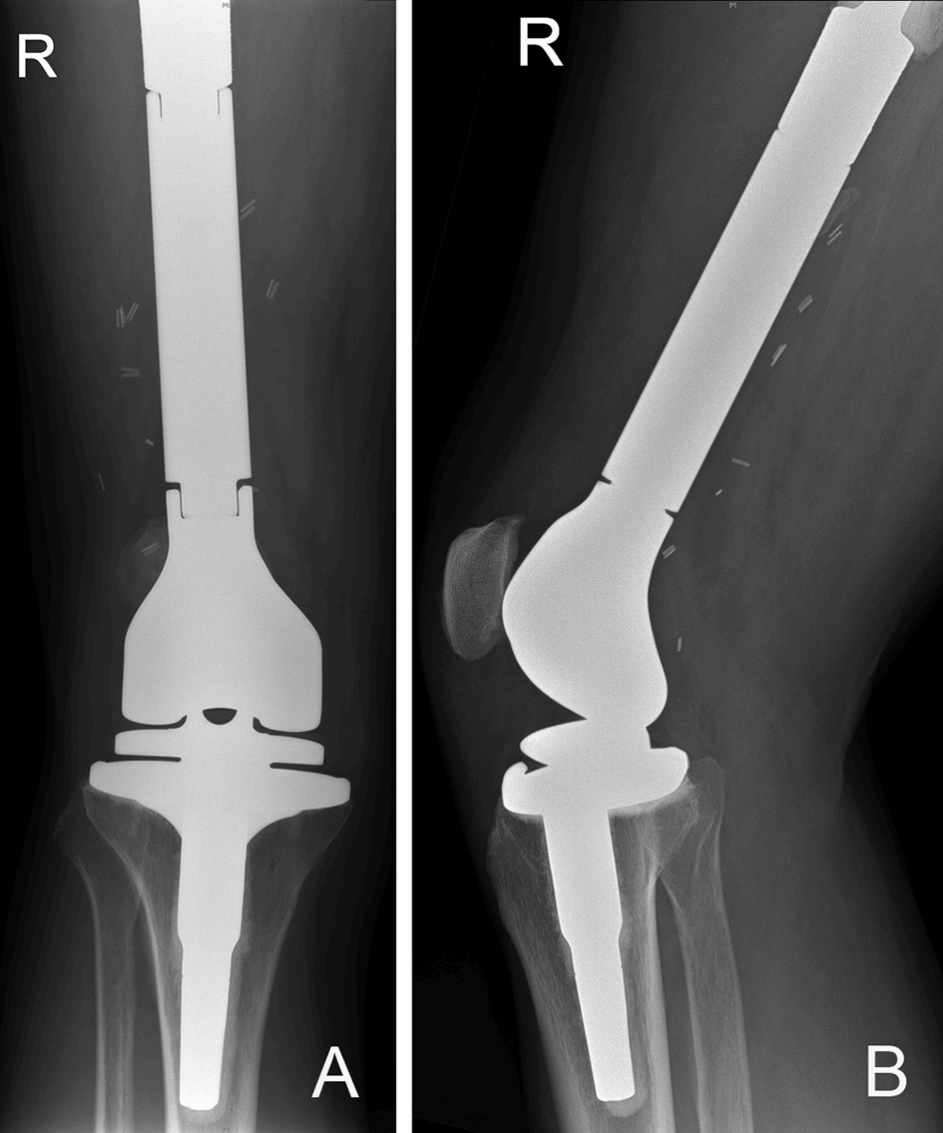
**(A)** AP and **(B)** lateral radiographs of a right distal femur modular endoprosthesis. The modularity of these implants allow for reconstruction of long segments of bone defects.

In cases where lesions involve a large part of the distal femur, resection, and reconstruction using a distal femoral replacement endoprosthesis is preferred (Figure 11). Guzik et al. reported their findings on 67 patients with metastatic bone disease who underwent radical resection and modular prosthesis replacement. They concluded that radical resection of the area affected by tumor followed by reconstruction using modular prosthesis provided patients with significant improvement in pain and function. They also concluded that radical resection of the tumor prevents local recurrence and future implant loosening (9).

### Humerus

Following the femur, the humerus is the second most common site for bony metastasis. As with the femur, the proximal region of the humerus is the most frequently affected area, followed by the diaphysis (44). Being a non-weight bearing bone, majority of traumatic humeral fractures are amenable to conservative treatment with acceptable outcome. This is not the case in the setting of metastatic bone disease, as healing without surgical intervention is less likely. A painful, non-united humeral pathological fracture has a significant negative impact on a patient's functional independence and quality of life (44).

For lesions involving the humeral head and metaphysis, replacement with an endoprosthesis using a long cemented stem has shown reliable results. Kumar et al. in their retrospective review of 100 patients who underwent proximal humerus endoprosthesis replacement, showed reasonable functional outcome with good implant survivorship (86.5% at 20 years). They found that the length of the resected bone segment affected the functional outcome (59). Of note, their study included patients who underwent proximal humerus resection and reconstruction for primary bone sarcoma rather than metastasis.

In selected cases where the lesion is still contained within reasonable bone stock, locking plate fixation with bone cement augmentation may be sufficient (10).

Intramedullary nails are frequently used for diaphyseal lesions or pathological fractures. The ability to insert intramedullary nails via a minimally invasive approach, minimizes intraoperative blood loss and operative time significantly. The other advantage of intramedullary nail over plate fixation is the ability to span the whole bone, which minimizes the risk of future periprosthetic fractures due to disease progression.

**Figure 12:**
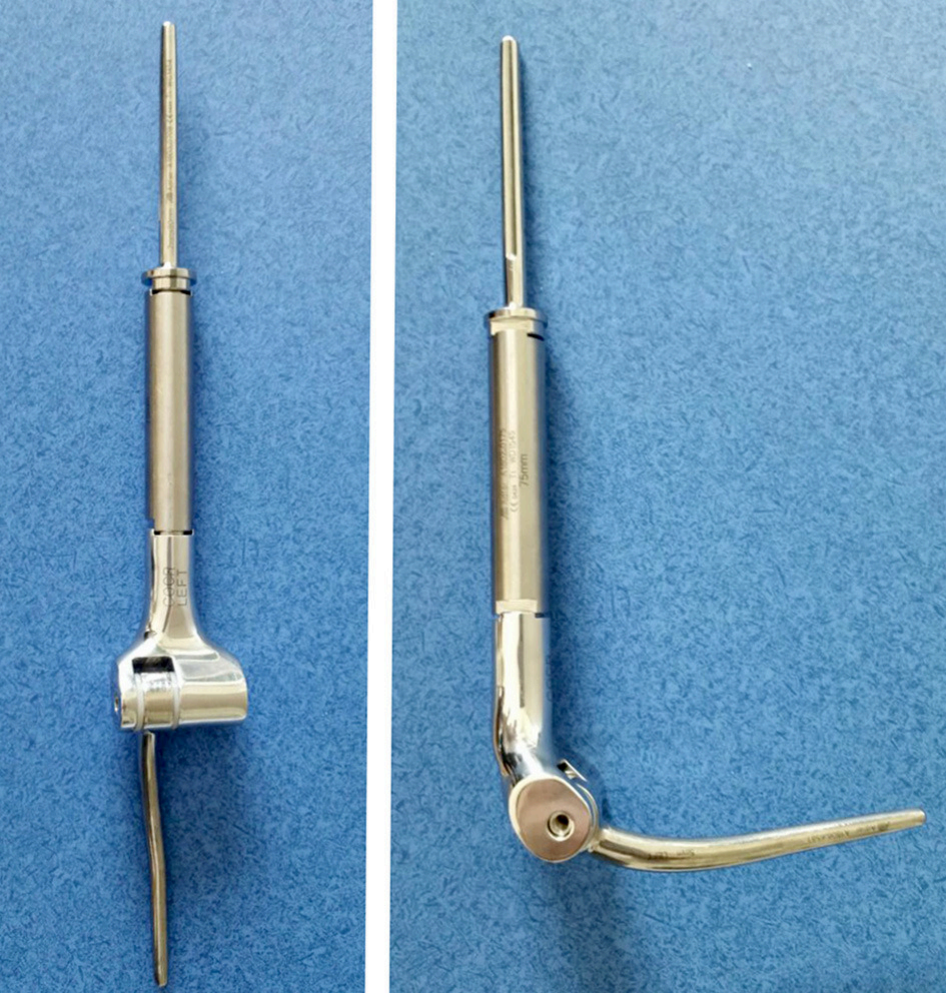
Modular total elbow endoprosthesis implant. These implants allow for reconstruction of a large segment of bone defect while preserving some elbow function.

Bone metastasis in the distal humerus can be challenging to manage. Distal periarticular lesions may require an elbow joint sacrificing procedure, such as local resection followed by reconstruction using a total elbow endoprosthesis (Figure 12) (60).

### Tibia

Although rare, involvement of the tibia in metastatic bone disease can have a major impact on patient's mobility and quality of life. Resection of extensive proximal tibial metastasis with endoprosthesis reconstruction is a viable option, however careful planning is required, as resection around this region is associated with high rates of wound complications, often requiring additional soft tissue coverage procedures. In smaller lesions where there is no joint involvement, the option of locking plate fixation with bone cement augmentation may suffice (2). As with the femur and the humerus, diaphyseal lesions are best treated with locked intramedullary nails. This usually provides significant pain relief and allows early weight bearing (61).

The options for addressing lesions involving the distal tibia or ankle joint are more limited. Fixation using locking plates with cement augmentation may be suitable for extraarticular involvement, however involvement of the ankle joint usually requires a below knee amputation (62).

### Pelvis

**Figure 13:**
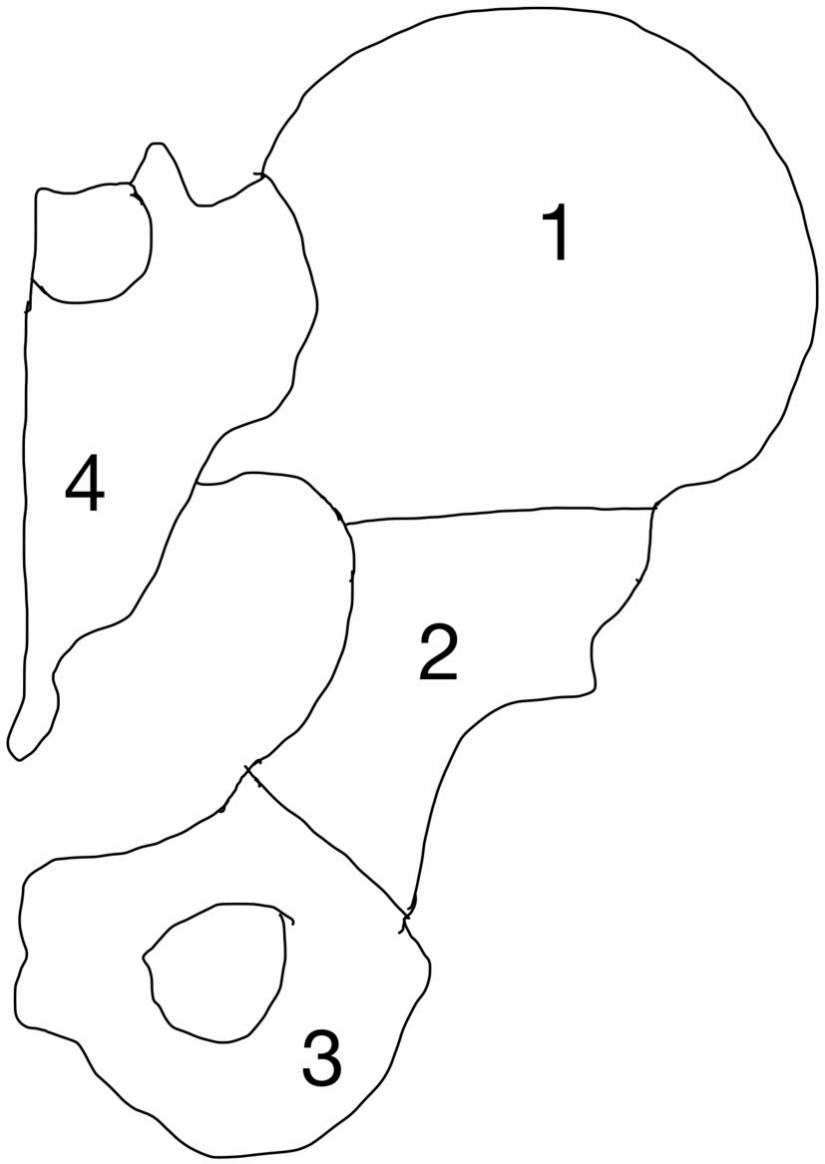
Enneking classification system of the pelvis (Zone 1–4).

The pelvis and spine are the most common sites affected by metastases (6, 63–65). The pelvic region undergoes significant amounts of mechanical stress, which predisposes it to pathological fractures in the setting of bone metastasis. Surgical treatment of pelvic metastases can be challenging because of its complex bony anatomy and neighboring vital structures. Enneking et al. devised a classification system to divide the pelvis into four anatomic regions (Figure 13). This classification system was developed to provide a commonality of language when describing pelvic tumors and location of surgery (63).

Zone 1 and 3 are non-weight-bearing zones, whereas zone 2 is the articular zone through which weight bearing occurs, and zone 4 is where stress transfer occurs between the spine and the pelvis. Of note, although Zone 1 is not directly involved in weight bearing, it is an important part of the stress transfer zone in the pelvis. Fractures may occur anywhere in the pelvis but the periacetabular region (zone 2) is the most vulnerable due to high mechanical stresses during weight bearing.

Harrington specifically classified metastases in relation to the acetabulum because of the importance of this anatomical structure. He described 4 types: Type 1 is where the subchondral bone of the acetabulum is still intact. Type 2 has medial wall involvement but an intact superior part (acetabular roof) and lateral wall. Type 3 has medial wall, lateral rim and acetabular roof involvement and Type 4 is when the acetabulum is collapsed completely (64).

Capanna et al. introduced an algorithm that divided patients into 4 classes (Table 3) based on the nature of the metastatic disease and its location (6).

**Table 3:** Capanna classification.

**Capanna class**	**Pelvis**
Class 1	Solitary metastatic lesion Primary with good prognosis Interval over 3 years since detection of primary tumor
Class 2	Pathological fracture in the periacetabular region
Class 3	Supra-acetabular osteolytic lesion
Class 4	Multiple osteoblastic lesions at all sites Osteolytic or mixed lesions in iliac wing and anterior pelvis Small periacetabular osteolytic lesions

Muller and Capanna published a guideline for the surgical treatment of metastatic pelvic lesions, taking into consideration the Enneking and Harrington classification for acetabular defects (65).

All patients in Capanna class 1, 2, and 3 should be considered for surgical treatment. Patients in class 1 may be treated aggressively with curative intent. If the lesion is in zone 1 or 3, reconstruction may not always be necessary. For lesions in zone 2, reconstruction with prosthetic or biologic construct is required (64).

The option of treatment for patients in class 2 and 3 is to provide a durable construct, although surgery may not be performed with curative intent. The aim is to achieve a marginal or intralesional resection followed by reconstruction options according to the amount of the periacetabular bone loss. Harrington Type 1 defects are usually addressed by curettage and cementation or conventional arthroplasty. In Type 2 defects, where there is medial acetabular wall involvement, joint replacement with the use of reinforcement ring is necessary. In Type 3 defects, total hip replacement with cementation of bone defects reinforced with transosseous pins is the recommended surgical option. In Type 4 defects, the options include pelvic megaprosthesis, saddle femoral prosthesis or massive allograft with joint replacement.

Patients in Capanna class 4 should be treated conservatively (chemotherapy, radiotherapy or hormonal therapy). The aim of treatment in this class is to palliate pain in order to improve quality of life (65).

**Figure 14:**
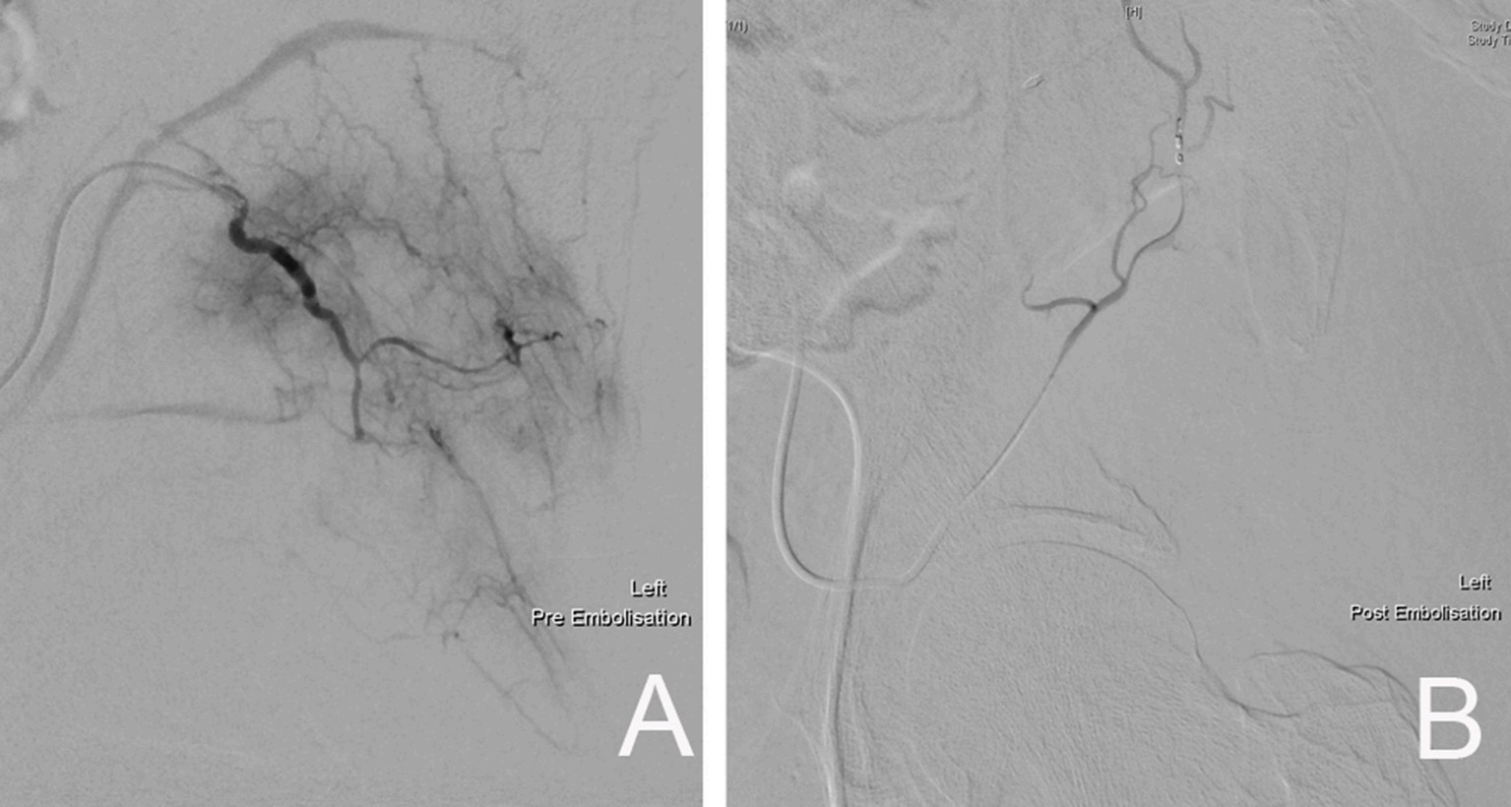
**(A)** Pre-embolization angiography demonstrating the rich blood supply to the left pelvic metastatic lesion. **(B)** Post-embolization fluoroscopic image showing complete devascularization of the metastatic lesion.

In dealing with highly vascular metastatic lesions, such as that from renal and thyroid carcinoma, it is recommended that preoperative angiographic selective arterial embolization be performed in order to minimize intraoperative blood loss (Figure 14) (66–68). Chatziioannou et al. conducted a retrospective study on the effectiveness of preoperative embolization in bone metastasis from renal cell carcinoma. Their study included 28 preoperative embolization procedures, which were divided into those with complete and incomplete revascularization of lesions post-embolization. Their findings show that complete devascularization of metastatic lesions resulted in significantly less intraoperative mean blood loss (535 ± 390 vs. 1.247 ± 1.047 ml) and transfusion requirements (1.3 ± 1 vs. 2.4 ± 1.2 units). They highlighted the importance of embolizing every feeder vessel to the metastatic lesion to achieve complete devascularization, since an incomplete result significantly increased intraoperative blood loss and transfusion requirements (67).

## Adjunctive management

### Radiation therapy

Radiation therapy plays an important role in the treatment of skeletal metastasis, both as an adjunct to other treatments and as monotherapy (69). Its uses have been shown to be effective in reducing pain, preventing pathological fractures and minimizing the need for further surgery (70).

Radiation therapy is commonly administered as a single or multiple fraction therapy. The type of tumor and the general condition of the patient usually dictates which method of radiation therapy is to be administered (69, 70). De Felice et al. suggested that in uncomplicated painful bone metastases, a single fraction of 8 Gy for three-dimensional conformal radiation therapy (3D-CRT) or 15–24 Gy stereotactic body radiation should be given; in cases of pathological fractures, the same authors suggested 5 fractions of 20 Gy or 10 fractions of 30 Gy for 3D-CRT to be administered (69). Lutz et al. (71) in their ASTRO Evidence-Base Guideline in 2011, update in 2016, recommended a single dose of 8 Gy fraction for targeted bone lesion. Should radiation therapy be deemed necessary as a post-operative adjunct, they suggested the use of multifractionated radiation therapy over single-fraction therapy. They concluded that the need for re-irradiation in those undergoing single-fraction therapy is up to 20% in contrast to only 8% in those who received multi-fraction therapy (72).

Despite its effectiveness as a treatment modality in the treatment of metastatic bone disease, it is important to consider the dose-dependent toxicity associated with radiation therapy. Both systemic and local side effects have been reported (2–40%), which may include nausea, vomiting and local soft tissue generated pain (69, 70). The presence of multiple symptomatic metastases and the proximity of the metastases to critical structures may render radiation therapy unsuitable in certain cases (73).

### Antiresorptives

Antiresorptive therapies are commonly used for the treatment of osteoporosis. The five main classes of antiresorptives used clinically include: Bisphosphonates, estrogens, calcitonin, selective estrogen receptor modulators (SERMs), and monoclonal antibodies such as Denosumab.

Bisphosphonates are potent inhibitors of osteoclast-mediated bone resorption (74, 75). In recent years it has become standard of treatment for lytic lesions, such as found in multiple myeloma and breast cancer (75). Bisphosphonate use in the setting of metastatic bone disease has been shown to cause recalcification of lytic metastasis (74, 75), which in turn reduces pain and minimizes the development of further lesions (76). Some of the most common Bisphosphonates used include Zolendronic acid, Clodronate, and Pamidronate (77, 78).

Bisphosphonates, in particular Zolendronic acid has been shown to have anti-tumor effects through the inhibition of tumor cell proliferation, induction of apoptosis, inhibition of angiogenesis and other important effects (79–81). Terpos et al. in their recent analysis comparing Bisphosphonates vs. either placebo or no treatment, demonstrated that the use of Bisphosphonates in the treatment of patients with multiple myeloma had reduced the rate of pathological fractures. They also concluded that Zolendronic acid appeared to be superior when compared to other Bisphosphonates (76).

O'Carrigan et al. reviewed 44 randomized controlled trials which included 37.302 patients with breast cancer. Included were patients with early breast cancer, advanced breast cancer without metastasis and those with metastatic disease. They compared the effects of Bisphosphonates to placebo, other Bisphosphonates, other antiresorptive agents, and also examined the effect of early versus delayed treatment with Bisphosphonates. They concluded that in patients with early breast cancer, Bisphosphonates reduced the risk of bone metastasis and improved overall survival when compared to placebo or no treatment. In patients who have metastatic disease, Bisphosphonates were found to reduce the risk of skeletal related events (SRE) and appeared to reduce bone pain when compared to placebo or no Bisphosphonates (82).

In breast cancer, the role of Bisphosphonates has been well established, however there is a lack of consensus regarding the duration of treatment and whether all metastatic breast cancer patients should receive Bisphosphonates. Hillner et al. in their American Society of Clinical Oncology guideline on the role of Bisphosphonates in breast cancer, acknowledged that the duration of treatment is not well defined, however reported that the majority of patients tolerated treatment beyond 2 years. They recommended that once treatment is commenced, it should be continued until there is a decline in patient's performance status. They also concluded that patients who have multiple painful metastasis and metastases to weight-bearing bones, should be commenced on Bisphosphonates (83).

Denosumab is a fully human IgG2 monoclonal antibody that blocks RANKL with subsequent reduction in osteoclastic bone resorption, giving a Bisphosphonate-like action. Numerous studies have demonstrated that the use of Denosumab in metastatic bone disease have significantly reduced the development of skeletal-related events associated with bone metastases (84–86). Recent studies have also shown that blocking RANKL action on tumor cells had an inhibitory effect on tumor cells in *in vitro* and animal models, although the exact mechanism is not fully understood (87, 88). Gonzalez-Suarez et al. published their study on the role of RANKL on RANK expressing tumor cells in mice. They demonstrated that the inhibition of RANKL in breast cancer had resulted in a decrease in associated lung metastasis (89).

## Conclusion

Despite advances in medical treatment in cancer and the steady improvement in overall survival of cancer patients, the management of metastatic bone disease remains challenging. The treatment of metastatic bone disease is multi-modal and often includes a combination of medical therapy, radiation therapy, or surgery.

Advances in modern medical diagnostic imaging have allowed earlier detection of bone metastasis in the course of disease, enabling treating surgeons to intervene before pathological fractures occur. A vast array of implants and treatment options are available in our current modern orthopedic surgery arena, and these enhance the role of orthopedic surgeons in decision making when considering the best surgical treatment strategy. The goal of surgical treatment is to alleviate pain, restore function, and ultimately improve the quality of life of patients. The complexity of the management of patients with metastatic bone disease mandates a multidisciplinary approach with careful planning, in order to achieve the best and safest outcome for patients.

## Author's note

All deidentified images used in figures are from the image database of St. Vincent's Hospital, Melbourne, Australia.

## Author contributions

HS, LP, and PC: article conception and structuration. HS and LP: literature search and manuscript drafting. HS, LP, and PC: critical revision of manuscript content. PC: approving final version of manuscript.

### Conflict of interest statement

The authors declare that the research was conducted in the absence of any commercial or financial relationships that could be construed as a potential conflict of interest.
